# Correction: The association between platelet-to-albumin ratio and diabetic peripheral neuropathy: A cross-sectional study in the Chinese population

**DOI:** 10.1371/journal.pone.0351559

**Published:** 2026-06-10

**Authors:** Wenting Deng, Siqi Zhang, Yueyang Zhang, Qin Wan

In the Funding statement, the grant number from the funder General Program of Science and Technology Department of Sichuan Province is listed incorrectly. The correct grant number is: 2024NSFSC0579.
