# Supplementary material for: The association between platelet-to-albumin ratio and diabetic peripheral neuropathy: A cross-sectional study in the Chinese population
Source: PLoS One. 2026 May 20;21(5):e0348402. doi: 10.1371/journal.pone.0348402 (PMC13189351; doi:10.1371/journal.pone.0348402)
Supplement: S1 Table — Cut-off indicates the optimal threshold value determined by the Youden index. AUC represents the area under the ROC curve, with corresponding 95% confidence intervals (CI). Sensitivity and specificity were calculated at the optimal cut-off point. ALB, serum albumin; PLT, platelet count; PAR, platelet-to-albumin ratio; PHR, platelet-to-high-density lipoprotein cholesterol ratio. (DOCX) [file pone.0348402.s002.docx]

| Variables | Cut-off | AUC | 95% CI | P value | Sensitivity | Specificity |
| --- | --- | --- | --- | --- | --- | --- |
| ALB | 50.05 | 0.413 | 0.378 – 0.448 | <0.001 | 58.3 | 95.7 |
| PLT | 216.5 | 0.581 | 0.546 – 0.615 | <0.001 | 48.8 | 64.4 |
| PAR | 5.5971 | 0.602 | 0.568 – 0.636 | <0.001 | 35.9 | 81.7 |
| PHR | 234.1717 | 0.542 | 0.507 – 0.577 | 0.017 | 29.2 | 78.8 |

S1 Table. Receiver operating characteristic (ROC) analysis of PAR and related indicators for diabetic peripheral neuropathy.

Cut-off, optimal cutoff value based on the Youden index; AUC, area under the receiver operating characteristic curve; CI, confidence interval; ALB, albumin; PLT, platelet count; PAR, platelet-to-albumin ratio; PHR, platelet-to-high-density lipoprotein cholesterol ratio.
