# Supplementary material for: The association between platelet-to-albumin ratio and diabetic peripheral neuropathy: A cross-sectional study in the Chinese population
Source: PLoS One. 2026 May 20;21(5):e0348402. doi: 10.1371/journal.pone.0348402 (PMC13189351; doi:10.1371/journal.pone.0348402)
Supplement: Attachment — Submitted filename: comments.docx [file pone.0348402.s003.docx]

Review Comments on "*The Correlation between Platelet-to-Albumin Ratio and Diabetic Peripheral Neuropathy: A Cross-Sectional Study in the Chinese Population*"

**Existing Issues and Improvement Recommendations**

1. **Improvement and Supplementation of Limitations in Study Design:**

Limitations of Cross-Sectional Design in Causal Inference: The current study can only confirm the correlation between PAR and DPN, but cannot clarify the causal relationship (e.g., whether elevated PAR is a cause or a result of DPN). It is recommended to further emphasize this limitation in the discussion section.

2. **Optimization of Data Interpretation and Statistical Analysis:**

Objective Evaluation of PAR Predictive Efficacy: ROC curve analysis shows that the AUC of PAR is 0.602, with a sensitivity of only 35.9%. Although the specificity reaches 81.7%, the overall predictive efficacy is moderately low, and its clinical utility as a single screening indicator for DPN is limited. It is recommended to objectively analyze this deficiency in the discussion, and propose that PAR be combined with other indicators (such as HbA1c, LDL-C, and other proven DPN risk factors) to construct a prediction model, so as to improve the screening efficacy; at the same time, supplement the possible reasons for the low sensitivity (e.g., the complex pathogenesis of DPN, and a single inflammation-related indicator is difficult to fully cover).

Completeness of Confounder Adjustment: Multivariate regression analysis has adjusted for a number of confounding factors, but it is not clear whether indicators directly related to DPN, such as "history of diabetic foot" and "results of nerve conduction velocity testing", are included (although patients with severe diabetic foot complications are excluded, mild to moderate lesions may still have an impact). It is recommended to supplement the basis for the selection of confounding factors (e.g., based on literature reports, results of univariate analysis, etc.), and verify whether the association between PAR and DPN remains stable after including the above potential confounding factors.

In-depth Exploration of Subgroup Analysis: Subgroup analysis shows that factors such as age, gender, and BMI have no interaction effect, but subgroup stratification analysis based on "PAR quartile grouping" (e.g., differences in the risk of DPN in each subgroup under different PAR levels) has not been conducted. It is recommended to supplement this analysis to further clarify whether the stability of the association between PAR and DPN is affected by PAR levels.

3. **Improvement of Details in Methodology and Result Presentation:**

Clarification of PAR Calculation Method: The study does not detailedly explain the calculation method of PAR (e.g., the unit of platelet count, the unit of albumin, and whether it is "platelet count (×10⁹/L) / albumin (g/L)"). It is recommended to clarify the specific calculation formula and unit of PAR in the methodology section to avoid misunderstandings by readers.

Verification of the Accuracy of Table Data: There is an abnormal situation in the two groups of data for the "γ-GGT" indicator in Table 1 (45.35 (17.7, 44.9), 49.62 (16.3, 42.9)), where the upper limit of the interquartile range is lower than the median, which may be an input error. It is recommended to verify the accuracy of all table data, correct abnormal values, and ensure data reliability.

Standard Use of Abbreviations: When "PAR", "DPN", and "T2DM" first appear in the abstract, their full names are indicated. However, the full names of some abbreviations (such as "ACR" and "PHR") are not specified when they first appear in the main text. It is recommended to standardize this uniformly and indicate the full names of all abbreviations when they first appear.

Unification of Reference Formats: The formats of some references are inconsistent (e.g., some lack DOI numbers, and the abbreviations of authors' names are not unified). It is recommended to unify and standardize them in accordance with the reference format requirements of PLOS ONE to ensure the accuracy of citation formats.

Clarity of Figures and Supplementary Explanations: The OR values and 95% CIs in Figure 2 (subgroup analysis forest plot) are not clearly presented. It is recommended to optimize the layout of the figure; the ROC curve in Figure 3 does not mark the confidence intervals of each indicator. It is recommended to supplement the 95% CIs of the AUC to fully demonstrate the predictive efficacy.
