# Supplementary material for: The association between platelet-to-albumin ratio and diabetic peripheral neuropathy: A cross-sectional study in the Chinese population
Source: PLoS One. 2026 May 20;21(5):e0348402. doi: 10.1371/journal.pone.0348402 (PMC13189351; doi:10.1371/journal.pone.0348402)
Supplement: Attachment — Submitted filename: Response to Reviewers.docx [file pone.0348402.s005.docx]

**Manuscript ID: PONE-D-25-53697**

**Manuscript:** *The association between Platelet-to-albumin ratio and Diabetic peripheral neuropathy: A cross-sectional study in the Chinese population*

We sincerely thank the Academic Editor and the reviewers for their thorough and constructive comments. We have carefully revised the manuscript in response to all points raised, . All page and line numbers reported below refer to the “Revised Manuscript with Track Changes”, text modifications are marked in red font, unless otherwise specified.

**Response to the Academic Editor**

Dear Academic Editor,

We sincerely thank you for your careful evaluation and constructive comments. We have revised the manuscript thoroughly to address every point raised. Below, we provide a detailed point-by-point response, and all changes are reflected in the tracked-changes version.

**Comment 1: “Correlation is a statistical term. Please change with association.”**

We appreciate this important comment. We agree and have replaced “correlation” with “association” in the title and throughout the manuscript, where we refer to the relationship between PAR and DPN as an epidemiologic association (rather than implying causality or a purely correlational relationship). (see Revised Manuscript with Track Changes, page 1, lines 1; page 2, lines 37;).

**Comment 2: “The Methods section in the abstract miss important information: when and how the study was conducted, which was the eligible population, which were the inclusion and exclusion criteria etc.”**

Thank you for this helpful comment. We revised the **Abstract—Methods** to include: (1) study design (cross-sectional), (2) data source (National MMC), (3) study time window (June 2018–December 2023 enrollment), (4) key inclusion/exclusion criteria, and (5) core analytic approach (logistic regression, subgroup analysis, restricted cubic spline, ROC).(Revised Manuscript with Track Changes **Abstract—Methods**: “This cross-sectional study included adult patients with T2DM enrolled in the national metabolic management center (MMC) database between June 2018 and December 2023. Eligible participants were selected according to predefined inclusion and exclusion criteria and were divided into diabetic peripheral neuropathy (DPN) and non-diabetic peripheral neuropathy (non-DPN) groups. The association between PAR and DPN was evaluated using logistic regression models, with subgroup analyses and restricted cubic spline regression to explore potential effect modification and dose–response relationships. Receiver operating characteristic (ROC) curve analysis was performed to assess the discriminative performance of PAR for DPN prevalence. ”See **Revised Manuscript with Track Changes**, page 2, lines 28–36).

**Comment 3: “The conclusion is too strong for the reported results.”**

Thank you for this important comment.We agree and have **softened the Conclusion** to avoid implying screening/prediction utility. The revised text emphasizes that PAR is **associated with the prevalence of DPN** and this association may be related to underlying inflammatory processes involved in DPN. (See **Revised Manuscript with Track Changes**, page 3, lines 62–64; page 29, lines 855–859;).

**Comment 4 : “Ethics statement is incomplete. Provide the data for ethics approval. Since the study "utilizes a registered dataset from the National Metabolic Management Centre (MMC)" it is unclear how participants gave the informed consent.” and “Provide the date for ethics approval.”**

Thank you for this important comment. We revised the **Ethics Approval** section to explicitly state that:

- all participants provided **written informed consent**,
- the study was approved by the ethics committee of the Affiliated Hospital of Southwest Medical University (**approval code: 2018017; approval date: February 2018**),
- the dataset used for analysis was **de-identified** prior to statistical analysis.

(**Revised Manuscript with Track Changes:**

“Each participant provided written informed consent related to participation in this study. The study was conducted according to the ethical guidelines of the 2013 2024 Declaration of Helsinki (latest revision) and was approved by the ethics committee of the Affiliated Hospital of Southwest Medical University (ethical approval code: 2018017, date: February 2018). ” See **Revised Manuscript with Track Changes**, page 6, lines152–156).

**Comment 5: “End the Introduction section with the aim of the study.”**

Thank you for this helpful comment. We revised the final paragraph of the **Introduction** to end with a clear, single-sentence aim describing the study objective: “Therefore, this study aimed to investigate the association between platelet-to-albumin ratio (PAR), a composite marker reflecting inflammatory and nutritional status, and the prevalence of diabetic peripheral neuropathy among patients with type 2 diabetes mellitus in a Chinese population.” (See **Revised Manuscript with Track Changes**, page 5, lines 111–114).

**Comment 6: “The methods must be described in such details to allow reproduction/replication. Please find some example for improving this section: "systematically curated by healthcare professionals" how? how many? when (prior to the survey or after?), "Samples that met the specified criteria were extracted from the MMC database from June 2018 and November 2022 and subsequently divided into two groups: DPN and non-DPN." we are in 2025 (almost 2026) please explain the gap. When the data were retrieved? Is the database publicly available? Provide details for VPT test (who applied the test? which training they have? etc.). It sound to be sex not gender. List "other pertinent factors". "patient's height and weight were also recorded" were measured or just recorded? "measured on the right arm using a standard mercury sphygmomanometer" when? (in the morning? in afternoon? etc.)"Analysis was adjusted for several factors." How were the factors selected? Why diabetes onset is not aa factor used in adjustement?”**

We sincerely thank you for your careful evaluation and constructive comments. We substantially expanded the **Methods** to improve reproducibility:

1. **Data collection personnel and procedure**

Thank you for this comment. We clarified that data was collected by **trained endocrinology physicians and nurses**, following standardized operating procedures, with **uniform training** before data collection.(Original text: “The National Metabolic Management Centre 118 database is a national, registered clinical management system with restricted access, and all data used in the present study were de-identified before analysis [22–24]. Data were collected by trained endocrinology physicians and nurses at participating centers following standardized operating procedures, and all personnel received uniform training on study-specific questionnaires and outcome measures before data collection.” See **Revised Manuscript with Track Changes**, page 5, lines 118–124).

2. **Explain the time gap**

Thank you for this comment. We clarified that **data extraction for the present analysis was conducted in December 2023** using the complete MMC database available at that time. From this database, we retrospectively identified patients enrolled between June 2018 and November 2022 according to predefined inclusion and exclusion criteria. Therefore, the time gap reflects the retrospective nature of data selection rather than delayed data collection. (Original text: “Data extraction was performed in December 2023 from the MMC database. Patients enrolled between June 2018 and November 2022 who met the predefined inclusion criteria were retrospectively identified and subsequently divided into diabetic peripheral neuropathy(DPN) and non-DPN groups for the present analysis.” See **Revised Manuscript with Track Changes**, page 6, lines 145–149).

3. **Database availability**

Thank you for this important comment. We clarified that the MMC database is a national, real-world clinical management system with restricted access. As described in previous MMC-related publications, including large multicenter studies, the database contains sensitive clinical and longitudinal health information collected under ethical approval and specific informed consent, which does not permit unrestricted public data sharing. The MMC data governance committee therefore governs data access. In line with established MMC publications, the dataset used in the present study cannot be made publicly available, but de-identified data may be provided upon reasonable request subject to appropriate approval.(see **Revised Manuscript with Track Changes**, page 5, lines 118–124; page 32, lines 935–942;).

4. **VPT/monofilament testing details**

Thank you for this comment. We expanded the DPN assessment description and clarified that VPT and monofilament testing were performed by **trained endocrinologists or certified technicians** who received standardized training and followed uniform procedures across centers.(Original text: “Diagnosis of diabetic peripheral neuropathy was based on standardized clinical criteria. All assessments were performed by trained endocrinologists or certified technicians who had received uniform training in neuropathy evaluation before the study and who followed standardized operating procedures across participating centers. Patients were required to have a confirmed diagnosis of type 2 diabetes mellitus, with neuropathic symptoms occurring at or after diagnosis of diabetes.” see **Revised Manuscript with Track Changes**, page 6-7, lines 162–176).

5. **Sex vs gender**

Thank you for this comment. We replaced “gender” with **“sex”** throughout the manuscript to ensure correct terminology.[see **Revised Manuscript with Track Changes**, page 8, lines 315; page 9, lines 392; page 10, lines 415; page 12, lines 458(Table1: Row 2 and Column 1); page 15, lines 480; page 16, lines 492(Table2: Row 5 and Column 1); page 18, lines 507(Table3: Row 3 and Column 1);page 21, lines 518; page 22, lines 523(Table4: Row 9-10 and Column 1);page 22, lines531;page 39, lines 1132].

6. **“Other pertinent factors”**

Thank you for this comment. We clarified that other sociodemographic variables (e.g., occupation, residence, education) recorded in MMC but were **excluded from** the present analysis because they were not directly relevant to the study objectives. We have deleted “other pertinent factors”(Original text: “Clinical data were collected as follows: The patient's demographic information includes sex and age. ”See **Revised Manuscript with Track Changes**, page 8, lines 315).

7. **“Height and weight recorded”**

We appreciate this important comment. We revised wording to clarify that these were **measured** (not merely abstracted) during the standardized physical examination.(Original text: “The patients' height and weight were also measured. ” See **Revised Manuscript with Track Changes**, page 8, lines 317).

8. **Blood pressure measurement timing**

Thank you for this comment. We clarified BP was measured **in the morning** after ≥5 minutes rest, with three readings averaged. (Original text: “Blood pressure was measured in the morning after at least 5 minutes of rest. ” See **Revised Manuscript with Track Changes**, page 8, lines 317-318).

9. **Covariate selection and diabetes onset/duration**

Thank you for this comment. We clarified that covariates were selected a priori based on clinical relevance, the literature, and univariate analyses, rather than solely on baseline group differences. (Original text: “The analysis was adjusted for several factors, including age, sex, smoking, alcohol consumption, duration of diabetes, SBP, DBP, BMI, FPG, 2hPG, glycated hemoglobin (HbA1c), Hb, RBC, WBC, HCT, MCV, MCH, MPV, ALT, AST, ALP, γ-GGT, BUN, TG, TC, HDL-C, LDL-C, urinary ACR, Cr, and eGFR; covariates included in the multivariable models were selected based on a combination of prior literature, clinical relevance, and univariate analyses. ”See **Revised Manuscript with Track Changes**, page 10, lines 491–496).

We also explicitly addressed diabetes duration: it was included in the models and was determined from patient-reported time since diagnosis, collected via standardized questionnaires during visits/follow-up. (Original text: “Only participants with confirmed type 2 diabetes mellitus were included in the present analysis. Diabetes duration was determined based on patient-reported time since diagnosis, collected during standardized clinical visits and follow-up.” see **Revised Manuscript with Track Changes**, page 6, lines 142–146).

**Comment 7: “Check the latest updates of Declaration of Helsinki.”**

Thank you for this comment. We updated the ethics phrasing to reflect the manuscript’s stated compliance with the **latest revision** of the Declaration of Helsinki.(Original text: “The study was conducted according to the ethical guidelines of the 2024 Declaration of Helsinki (latest revision) and was approved by the ethics committee of the Affiliated Hospital of Southwest Medical University (ethical approval code: 2018017, date: February 2018).”See **Revised Manuscript with Track Changes**, page 6, lines 153–155).

**Comment 8: “Provide the date for ethics approval.”**

Thank you for this comment. We have provided the ethical date. For further details, please refer to **comment 4 and comment 7**.

**Comment 9: “In some places, the writing is telegraphic (e.g., "Diagnosis of peripheral neuropathy in type 2 diabetes:(1) 113 Have a clear history of"; Calculations). In section named Calculations please check for brackets”.**

Thank you for this comment. We revised telegraphic fragments into complete sentences (including the DPN diagnostic criteria section) and corrected formatting issues in the **Calculations** section (including bracket/format consistency and equation presentation).(Original text: “Diagnosis of diabetic peripheral neuropathy was based on standardized clinical criteria. All assessments were performed by trained endocrinologists or certified technicians who had received uniform training in neuropathy evaluation before the study and who followed standardized operating procedures across participating centers. Patients were required to have a confirmed diagnosis of type 2 diabetes mellitus, with neuropathic symptoms occurring at or after diagnosis of diabetes. Neurological symptoms, including numbness, pain (tingling or prickling, pricking, burning, or aching), and sensory abnormalities (abnormal cold or heat sensations, nociceptive hypersensitivity, and dysesthesia) in the toes, feet, legs, or upper limbs, were assessed by structured questioning. Bilateral Achilles tendon reflexes were examined in the knee standing position and recorded as present, diminished, or absent. The vibration perception threshold (VPT) was assessed at the metatarsophalangeal joint of the hallux using a neurothesiometer (Bio-Thesiometer; Bio-Medical Instrument Co., Newbury, OH, USA). The patients were first instructed on how to recognize the vibration sensation. The stimulus amplitude was gradually increased from zero, and the patients were asked to indicate when the vibration was first perceived. Measurements were performed three times on the plantar surface of each hallux, and the median value was recorded as the VPT for each foot. Sensitivity to touch was evaluated using a 5.07/10-g Semmes-Weinstein monofilament (SWM) at four standardized sites on each foot( three plantar sites and one dorsal side). The monofilament was applied perpendicular to the skin until it just buckled, and was maintained for approximately 2 seconds. Diabetic peripheral neuropathy was defined as the VPT T ≥ 25 V and/or inability to perceive the 10-g monofilament at one or more test sites”See **Revised Manuscript with Track Changes**, page 6-7, lines 162–192).

**Comment 10: “Define abbreviations the first time used in the body”.**

Thank you for this comment. We revised the manuscript to define all abbreviations at first occurrence in the main text (including items such as ACR, PHR, etc.).

**Comment 11: “Mean 56.21 and SD 50.65 indicates non-normal distribution.”**

Thank you for this comment. We clarified that values like **“56.21 (50, 65)”** are **median (IQR)**, not mean (SD). We revised table formatting and footnotes to clearly present non-normally distributed variables as median (IQR), avoiding misinterpretation.(see **Revised Manuscript with Track Changes**, page 12, lines 458(Table 1).

**Comment 12: “It is unclear how duration of diabetes was determined. It is also unclear if the evaluated cohort had or not any type of DM.”**

Thank you for this comment. We clarified that diabetes duration was determined based on patient-reported time since diabetes diagnosis, collected through standardized questionnaires during clinical visits and follow-up assessments. This approach is consistent with routine clinical practice and has been widely used in large-scale registry-based studies.(Original text: “Diabetes duration was determined based on patient-reported time since diagnosis, collected during standardized clinical visits and follow-up. ”see **Revised Manuscript with Track Changes**, page 6, lines 144-145).

The National Metabolic Management Centre (MMC) is a nationwide, multicenter standardized metabolic management platform that includes individuals with prediabetes as well as various types of diabetes. Participants are followed longitudinally with standardized assessments of metabolic health status. Once a diagnosis of diabetes is established during follow-up, patients are managed and followed according to standardized diabetes care pathways. In the present study, only patients with confirmed type 2 diabetes mellitus (T2DM), diagnosed according to the American Diabetes Association (ADA) Standards of Care in Diabetes, were included. Individuals without diabetes, as well as those with type 1 diabetes, gestational diabetes, or other specific types of diabetes, were explicitly excluded. (Original text: “Only participants with confirmed type 2 diabetes mellitus were included in the present analysis. ” See **Revised Manuscript with Track Changes**, page 6, lines 143-145).

**Comment 13: “Correlation analysis seems to have no contribution to the study since logistic regression is reported.”**

We appreciate the Academic Editor’s comment. We agree that logistic regression provides the primary evidence for the association between PAR and DPN. In the revised manuscript, we have repositioned the correlation analysis as an exploratory descriptive analysis intended to illustrate the general relationships between PAR and related clinical variables, and we have reduced its emphasis in Discussion section. (Original text: “We observed that PAR levels were higher in patients with DPN than in those without DPN, and correlation analysis showed a positive association between PAR and DPN-related variables (r = 0.171), suggesting a weak magnitude of association and limited clinical effect size. ”See **Revised Manuscript with Track Changes**, page 23-24, lines 561–591).

**Comment 14: “Looking to statistical analysis result that the aim of the manuscript is to show the readers the potential of statistical analysis. Please rethink analysis based on clinical reasoning. (think of the clinical relevance of an OR equal to 1.2 when 1 means the same risk in both groups). It is unclear how the adjustments were decided (for example, smoking, alcohol consumption, duration of diabetes proved no statistically significant differences between groups)”.**

Thank you for this comment. We revised the Discussion to interpret findings from a clinical perspective, explicitly acknowledging that the adjusted association is modest (OR ≈ 1.1–1.2) and does not indicate strong clinical utility as a standalone indicator. (Original text: “Platelet-to-albumin ratio (PAR) was modestly associated with the prevalence of DPN after adjustment (OR = 1.2). The association appeared more apparent at higher PAR levels, whereas weaker associations were observed at lower levels. From a clinical perspective, this modest association is not unexpected. DPN is a complex and multifactorial complication of diabetes, resulting from the cumulative effects of metabolic dysregulation, microvascular injury, oxidative stress, and chronic inflammation over time[39].”see **Revised Manuscript with Track Changes**, page 26, lines 720–726).

We also clarified that covariates were chosen based on clinical relevance and literature rather than baseline statistical differences; thus smoking, alcohol consumption, and diabetes duration were retained for appropriate confounding control. (Original text: “The analysis was adjusted for several factors, including age, sex, smoking, alcohol consumption, duration of diabetes, SBP, DBP, BMI, FPG, 2hPG, glycated hemoglobin (HbA1c), Hb, RBC, WBC, HCT, MCV, MCH, MPV, ALT, AST, ALP, γ-GGT, BUN, TG, TC, HDL-C, LDL-C, urinary ACR, Cr, and eGFR; covariates included in the multivariable models were selected based on a combination of prior literature, clinical relevance, and univariate analyses. ”See Revised Manuscript with Track Changes, page 10, lines 414–420).

**Comment 15: “Do not start a sentence with an abbreviation.”**

Thank you for this comment. We reviewed the manuscript and revised sentences that began with abbreviations (e.g., “PAR…”, “DPN…”) by spelling out the full term or restructuring the sentence.

**Comment 16: “Do not duplicate information along the manuscript "The study included 1141 patients with T2DM, who were divided into the DPN group (n=714) and the non-DPN group (n=427)." Although r=0.171, P<0.001 is statistically significant tell nothing from clinical point of view.”**

Thank you for this comment. We removed repeated sample-size/group statements from multiple sections and retained them only where appropriate (Study Population/flow diagram). We also revised interpretation of r=0.171 to indicate weak association and limited clinical meaning explicitly. (Original text: “We observed that PAR levels were higher in patients with DPN than in those without DPN, and correlation analysis showed a positive association between PAR and DPN-related variables (r = 0.171), suggesting a weak magnitude of association and limited clinical effect size. ”see **Revised Manuscript with Track Changes**, page 24, lines 561–588).

**Comment 17: “The discussion is based on statistical significance and it is too strong for the reported results. Your statistical analysis looks like fishing and lack clinical reasoning.”**

**Comment 18: “Discuss the reported results from clinical point of view.”**

**Comment 19: “"PAR’s moderate predictive value (AUC = 0.602)" this is poor not moderate. "and further multicenter and longitudinal studies are needed to validate this conclusion" As the results looks like, no other studies are needed.”**

We agree with the Academic Editor’s comment and have revised the Conclusion accordingly. We acknowledge that some parts of the original Discussion placed excessive emphasis on statistical significance, which may have given the impression of overinterpretation or exploratory (“fishing”) analyses. In the revised manuscript, we have substantially revised the Discussion to adopt a more clinically grounded and cautious interpretation of our findings. Specifically, we have de-emphasized correlation coefficients and isolated statistically significant results, clarified that the observed association between PAR and DPN is modest in magnitude, and explicitly stated that PAR has limited clinical applicability as a standalone indicator given its poor discriminative performance. We further repositioned PAR as an associated marker reflecting inflammatory and nutritional status rather than a predictive or screening biomarker. Overall, the revised Discussion now focuses on clinical relevance, biological plausibility, and acknowledged limitations, thereby avoiding overstatement and reducing the risk of data-driven interpretation. (see **Revised Manuscript with Track Changes**, page 23-28, lines 558–853).

**Comment 20: “Discuss generalizability of the reported results.”**

Thank you for this comment. We added text discussing that this is a Chinese MMC-based cohort, which may limit external generalizability to other ethnicities/healthcare settings, and encourage cautious interpretation.(Original text: “In addition, the study population was derived from a Chinese cohort enrolled in a nationwide metabolic management program, which may limit the generalizability of the findings to other ethnic groups or healthcare settings.”See Revised Manuscript with Track Changes, page 28, lines 823–825)

**Comment 21: “No pathophysiological explanation of the results is presented in the Discussion section.”**

We thank you for this valuable comment. We expanded mechanistic discussion covering platelet activation/inflammation, hypoalbuminemia as an inflammatory state marker, and how PAR may reflect systemic inflammatory–nutritional milieu relevant to DPN, while clearly stating that mechanisms were not directly tested. (Original text: “Diabetic peripheral neuropathy (DPN) is a multifactorial complication of diabetes, in which chronic hyperglycemia, insulin resistance, obesity, and dyslipidemia collectively contribute to oxidative stress and low-grade systemic inflammation[28]. These metabolic disturbances activate several biological pathways, such as the protein kinase C, polyol advanced glycation end-products (AGEs), mechanistic target of rapamycin (mTOR), poly(ADP-ribose) polymerase (PARP), and mitogen-activated protein kinase (MAPK) pathways, ultimately leading to neuronal injury and apoptosis[9,29]. ” See **Revised Manuscript with Track Changes**, page 24-25, lines 606–640; “Metabolic abnormalities such as hyperglycemia, insulin resistance, obesity, and dyslipidemia contribute to elevated systemic inflammation and oxidative stress, which may promote platelet activation, adhesion, and aggregation [34,35]; Activated platelets release a range of inflammatory mediators that interact with immue cells, including lymphocytes and monocytes, thereby amplifying local inflammatory responses that may contribute to myelin damage and impaired nerve repair[36,37]. In parallel, hypoalbuminemia has been reported to be associated with adverse outcomes in several chronic conditions, including cardiovascular disease and diabetic complications.[40,41]. Pro-inflammatory cytokines such as interleukin-1β (IL-1β), interleukin-6 (IL-6), interleukin-8 (IL-8), and TNF-α have been linked to reduced serum albumin levels, suggesting that lower albumin may reflect an underlying inflammatory state [42,43]. In this study, the observed association between lower albumin levels and prevalent DPN is consistent with these observations. It supports the interpretation that albumin serves as an indirect marker of systemic inflammation rather than a direct causal factor in the development of neuropathy.” Page 26-27,lines 733–776).

**Comment 22: “Your results do not support the following statement "suggesting PAR as a simple, cost-effective, and accessible biomarker for DPN risk assessment".”**

We agree with the Academic Editor’s comment and have revised the Conclusion accordingly. The statement suggesting PAR as a simple, cost-effective biomarker for DPN risk assessment has been removed. The Conclusion has been rewritten to clarify that PAR shows a modest association with DPN and should be interpreted as an associated marker reflecting inflammatory and nutritional status. (Original text: “This study found that higher PAR was modestly associated with the prevalence of diabetic peripheral neuropathy in patients with type 2 diabetes mellitus. The platelet-to-albumin ratio (PAR) may reflect underlying inflammatory and nutritional disturbances associated with DPN and should be interpreted within a broader clinical and metabolic context.”see **Revised Manuscript with Track Changes**, page 29, lines 855–859).

**Comment 23: “Upload raw data when you submit your revision.”**

We are grateful for this comment from the Academic Editor. For a detailed explanation, please refer to point 3. Database Availability in Comment 6.

**Response to Reviewer #1**

We thank Reviewer #1 for constructive recommendations.

1. **Improvement and Supplementation of Limitations in Study Design:Limitations of Cross-Sectional Design in Causal Inference: The current study can only confirm the correlation between PAR and DPN, but cannot clarify the causal relationship (e.g., whether elevated PAR is a cause or a result of DPN). It is recommended to further emphasize this limitation in the discussion section.**

We thank the reviewer for this insightful and constructive comment. We agree and expanded the limitations section, explicitly stating that cross-sectional design precludes causal inference and that PAR may be a correlate rather than cause of DPN. (Original text: “First, the cross-sectional design precludes causal inference, and the observed association between PAR and DPN reflects prevalence rather than disease development over time.” See **Revised Manuscript with Track Changes**, page 28, lines 816–818).

2. **(1)Objective Evaluation of PAR Predictive Efficacy: ROC curve analysis shows that the AUC of PAR is 0.602, with a sensitivity of only 35.9%. Although the specificity reaches 81.7%, the overall predictive efficacy is moderately low, and its clinical utility as a single screening indicator for DPN is limited. It is recommended to objectively analyze this deficiency in the discussion, and propose that PAR be combined with other indicators (such as HbA1c, LDL-C, and other proven DPN risk factors) to construct a prediction model, so as to improve the screening efficacy; at the same time, supplement the possible reasons for the low sensitivity (e.g., the complex pathogenesis of DPN, and a single inflammation-related indicator is difficult to fully cover).**

We thank the reviewer for this comment. We agree that the discriminative performance of PAR alone is limited, as reflected by an AUC of 0.602 and a low sensitivity of 35.9% despite relatively high specificity. In the revised Discussion, we have explicitly acknowledged this limitation and clarified that PAR has poor predictive performance and limited clinical utility as a standalone screening indicator for DPN. (Original text: “Second, although PAR was statistically associated with DPN, the magnitude of this association was modest, and its discriminative performance was poor when evaluated alone, as indicated by a low area under the receiver operating characteristic curve and limited sensitivity. ” See **Revised Manuscript with Track Changes**, page 28, lines 818–821).

We have further supplemented the Discussion to explain the potential reasons for the low sensitivity. Specifically, diabetic peripheral neuropathy is a complex and multifactorial complication involving metabolic dysregulation, microvascular injury, oxidative stress, and chronic inflammation. A single inflammation-related composite marker such as PAR is therefore unlikely to capture the full spectrum of pathogenic processes underlying DPN, which may account for its modest association and limited discriminative ability. (Original text: “From a clinical perspective, this modest association is not unexpected. DPN is a complex and multifactorial complication of diabetes, resulting from the cumulative effects of metabolic dysregulation, microvascular injury, oxidative stress, and chronic inflammation over time[39]. A composite marker such as PAR is therefore likely to capture only a limited component of this complex disease process, which may explain its weak association with DPN when assessed independently. Despite limited discriminative performance, elevated PAR may reflect underlying inflammatory and nutritional disturbances relevant to the disease context of DPN, and it should be interpreted as an associated marker rather than a screening or predictive tool. ”see **Revised Manuscript with Track Changes**, page 26, lines 723–732).

Regarding the suggestion to combine PAR with other established risk factors (e.g., HbA1c, LDL-C) to construct a prediction model, we acknowledge that such an approach may improve screening performance. However, the primary objective of the present study was to evaluate the association between PAR and the prevalence of DPN rather than to develop or validate a predictive model. PAR should be interpreted as an associated marker reflecting inflammatory and nutritional status rather than a screening or predictive biomarker. Future studies may explore the incremental value of PAR when integrated into multivariable risk models.

**(2)Completeness of Confounder Adjustment: Multivariate regression analysis has adjusted for a number of confounding factors, but it is not clear whether indicators directly related to DPN, such as "history of diabetic foot" and "results of nerve conduction velocity testing", are included (although patients with severe diabetic foot complications are excluded, mild to moderate lesions may still have an impact). It is recommended to supplement the basis for the selection of confounding factors (e.g., based on literature reports, results of univariate analysis, etc.), and verify whether the association between PAR and DPN remains stable after including the above potential confounding factors.**

We thank the reviewer for this valuable comment. We clarified covariate selection as literature- and clinically driven. Variables such as diabetic foot history and nerve conduction outcomes were not included as “confounders” because they are typically **downstream manifestations or consequences** of neuropathy and could introduce over-adjustment/collider bias. We also note that severe diabetic foot complications were excluded, which reduces confounding from advanced foot disease.(method) (Original text: “Variables that represent clinical manifestations or consequences of diabetic peripheral neuropathy, such as a history of diabetic foot or nerve conduction study results, were not included as covariates, as they are downstream outcomes of neuropathy rather than independent confounding factors. Adjustment for such variables could lead to over-adjustment or collider bias.” See **Revised Manuscript with Track Changes**, page 10-11, lines 423-434).

**(3)In-depth Exploration of Subgroup Analysis: Subgroup analysis shows that factors such as age, gender, and BMI have no interaction effect, but subgroup stratification analysis based on "PAR quartile grouping" (e.g., differences in the risk of DPN in each subgroup under different PAR levels) has not been conducted. It is recommended to supplement this analysis to further clarify whether the stability of the association between PAR and DPN is affected by PAR levels.**

We thank the reviewer for this helpful suggestion regarding subgroup stratification by PAR levels. In response, we performed a quartile-based analysis of platelet-to-albumin ratio (PAR) to further evaluate whether the stability of the association between PAR and diabetic peripheral neuropathy (DPN) varied across different PAR levels. The results showed that the association was not uniform across quartiles: compared with the lowest quartile, a significantly increased odds of DPN was mainly observed in the highest PAR quartile, whereas lower and intermediate quartiles did not show statistically significant associations. (Original text: “The results of the subgroup analysis suggested that the association between PAR and DPN was not uniform across strata, as illustrated in Figure 2. When stratified by age, sex, body mass index (BMI), duration of diabetes, history of hypertension, HbA1c, and HDL-C levels, the highest PAR quartile (T4) was generally associated with higher odds of DPN compared with the lowest quartile (T1); however, this association did not reach statistical significance in all subgroups (Figure S1). Significant associations for T4 were observed in several strata, whereas in others the confidence intervals crossed unity. Nevertheless, interaction testing indicated that none of the examined subgroup variables significantly modified the association between PAR and DPN (all P for interaction > 0.05).” See **Revised Manuscript with Track Changes**, page 22, lines 528–543, Supplementary figure: figure S1).

3. **Improvement of Details in Methodology and Result Presentation:**
(1) **Clarification of PAR Calculation Method: The study does not detailedly explain the calculation method of PAR (e.g., the unit of platelet count, the unit of albumin, and whether it is "platelet count (×10⁹/L) / albumin (g/L)"). It is recommended to clarify the specific calculation formula and unit of PAR in the methodology section to avoid misunderstandings by readers.**

We thank the reviewer for this comment. We have carefully reviewed and standardized all calculation formulas and units in the manuscript to ensure clarity, accuracy, and reproducibility. In addition to explicitly defining the PAR formula, we systematically checked and unified all other calculated indices, including BMI, PHR, ACR, and eGFR. Duplicate or inconsistent formula expressions were removed, and all units were standardized across the manuscript. (Original text: “Body mass index (BMI) was calculated as weight (kg) divided by height squared (m²). The platelet to serum albumin ratio (PAR) was calculated as platelet count (10^9/L) divided by serum albumin concentration (g/L). The platelet-to-high-density lipoprotein cholesterol ratio (PHR) was calculated as platelet count (10^9/L) divided by HDL-C (mmol/L). Estimated glomerular filtration rate (eGFR) was calculated using the Modification of Diet in Renal Disease (MDRD) equation, incorporating serum creatinine concentration, age, and sex, as previously described.”See **Revised Manuscript with Track Changes**, page 9, lines 386–396).

(2) **Verification of the Accuracy of Table Data: There is an abnormal situation in the two groups of data for the "γ-GGT" indicator in Table 1 (45.35 (17.7, 44.9), 49.62 (16.3, 42.9)), where the upper limit of the interquartile range is lower than the median, which may be an input error. It is recommended to verify the accuracy of all table data, correct abnormal values, and ensure data reliability.**

We thank the reviewer for this comment. We checked all Table 1 entries and corrected any abnormal/inconsistent entries. The revised table now presents γ-GGT and other non-normal variables consistently as median (IQR). (see **Revised Manuscript with Track Changes**, page 13, table1:Row 23 and Column 1 ).

(3) **Standard Use of Abbreviations: When "PAR", "DPN", and "T2DM" first appear in the abstract, their full names are indicated. However, the full names of some abbreviations (such as "ACR" and "PHR") are not specified when they first appear in the main text. It is recommended to standardize this uniformly and indicate the full names of all abbreviations when they first appear.**

Thank you for this comment. We have amended the abbreviations "PAR", "DPN", and "T2DM" in the abstract. We reviewed the manuscript and revised sentences that began with abbreviations by spelling out the full term or restructuring the sentence.

(4) **Unification of Reference Formats: The formats of some references are inconsistent (e.g., some lack DOI numbers, and the abbreviations of authors' names are not unified). It is recommended to unify and standardize them in accordance with the reference format requirements of PLOS ONE to ensure the accuracy of citation formats.**

We thank the reviewer for this comment. We ensured abbreviations are defined on first use in the main text, aligned references to PLOS ONE formatting requirements. For further details, please refer to the references. (see **Revised Manuscript with Track Changes**, page33-38 lines 953-1112 ).

**Clarity of Figures and Supplementary Explanations: The OR values and 95% CIs in Figure 2 (subgroup analysis forest plot) are not clearly presented. It is recommended to optimize the layout of the figure; the ROC curve in Figure 3 does not mark the confidence intervals of each indicator. It is recommended to supplement the 95% CIs of the AUC to fully demonstrate the predictive efficacy.**

We thank the reviewer for this comment. We have revised Figure 2 to improve clarity by explicitly displaying the odds ratios (ORs) and corresponding 95% confidence intervals for each subgroup.We have supplemented the AUC values together with their corresponding 95% confidence intervals for all evaluated indicators. These results are summarized in **Supplementary Table 1**, which reports the AUC, 95% CI, optimal cut-off values, sensitivity, and specificity for each index, including PAR. (see figure2;see Supplementary documents: **Supplementary Table 1**).

**Response to Reviewer #2**

We thank Reviewer #2 for constructive recommendations.

1. **The cross-sectional design precludes causal inference, yet parts of the discussion and conclusions imply predictive or screening utility. Statements suggesting PAR as an “early screening biomarker” should be softened or reframed as risk association rather than prediction. I recommend to emphasize that PAR is associated with prevalent DPN, not predictive of future DPN.**

We thank the reviewer for this insightful and constructive comment. We agree and revised the Discussion and Conclusion to remove predictive language and consistently state that PAR is associated with prevalent DPN in a cross-sectional context. (Original text: “This study found that higher PAR was modestly associated with the prevalence of diabetic peripheral neuropathy in patients with type 2 diabetes mellitus. The platelet-to-albumin ratio (PAR) may reflect underlying inflammatory and nutritional disturbances associated with DPN and should be interpreted within a broader clinical and metabolic context.” see **Revised Manuscript with Track Changes**, page 29, lines 855–859, “First, the cross-sectional design precludes causal inference, and the observed association between PAR and DPN reflects prevalence rather than disease development over time.”page 28, lines 816–818).

2. **The ROC analysis shows an AUC of 0.602, which is generally considered poor discrimination, and sensitivity at the optimal cut-off is only 35.9%.Acknowledge more clearly that PAR alone has limited clinical applicability. Discuss whether PAR adds incremental value beyond established risk factors (such as HbA1c, duration of diabetes, ACR).**

We thank the reviewer for this comment. We revised text to describe AUC 0.602 as poor discrimination and acknowledge limited clinical applicability as a standalone marker. The present analyses focused on association rather than formal prediction modeling. Therefore, whether PAR provides incremental predictive value beyond established risk factors was not directly evaluated. Future studies employing dedicated risk prediction frameworks are needed to determine whether incorporating PAR can meaningfully improve risk stratification for DPN. (Original text: “Second, although PAR was statistically associated with DPN, the magnitude of this association was modest, and its discriminative performance was poor when evaluated alone, as indicated by a low area under the receiver operating characteristic curve and limited sensitivity. ” See Revised Manuscript with Track Changes, page 28, lines 818–821).

3. **At multiple points, DPN is reported as 427 cases, while elsewhere it is 714 cases. Carefully verify all sample sizes and ensure consistency across text, tables, and figures.**

We carefully checked and standardized group counts throughout the manuscript: non-DPN n=714 and DPN n=427 (total n=1141), and corrected any inconsistent statements in text/figures/legends.

We thank the reviewer for this comment. We carefully checked and standardized group counts throughout the manuscript: **non-DPN n=714** and **DPN n=427** (total n=1141), and corrected any inconsistent statements in text/figures/legends. (see **Revised Manuscript with Track Changes**, page 11, lines 445-446).

4. **Only the highest quartile (T4) shows a significant association. This threshold effect should be emphasized rather than implying a strong linear risk across all PAR levels. Clarify that clinically meaningful risk appears confined to higher PAR values, supported by quartile and spline analyses.**

We thank the reviewer for this comment. We revised the discussion to emphasize that the most evident clinically meaningful association is observed at higher PAR levels, consistent with quartile analysis (T4 significant) and spline analyses. (Original text: “This association remained after adjustment for a wide range of demographic, metabolic, hematological, and clinical covariates. Restricted cubic spline analyses supported an overall positive linear trend between PAR and DPN, without evidence of nonlinearity, indicating that the association is broadly consistent across the PAR distribution and more apparent at higher PAR levels. Subgroup analyses did not reveal significant effect modification across predefined subgroups. The findings from quartile-based analysis and restricted cubic spline modeling are complementary: although the spline analysis indicates an overall positive association between PAR and DPN, the quartile analysis suggests that statistically and clinically relevant risk is largely confined to individuals with higher PAR values. ”see **Revised Manuscript with Track Changes**, page 24, lines 591–601).

5. **The data availability statement indicates that data are available “upon reasonable request,” which does not fully comply with PLOS ONE data-sharing standards unless adequately justified.**

We thank the reviewer for this comment. We revised the Data Availability statement to provide a detailed justification: MMC data include sensitive human clinical information governed by MMC data policy and ethics/informed consent constraints. We state that de-identified data may be available upon reasonable request subject to required approvals, aligning with PLOS allowances for ethically restricted data. (Original text: “The data used in this study were obtained from the National Metabolic Management Centre (MMC), a nationwide, registered clinical management system in China. The dataset contains sensitive clinical data and potentially identifiable patient information collected under ethical approval and specific informed consent for clinical management and research purposes. Therefore, the data are not publicly available due to privacy and ethical restrictions imposed by the MMC data governance policy. De-identified data may be made available from the corresponding author upon reasonable request and subject to approval by the MMC data governance committee.”See **Revised Manuscript with Track Changes**, page 32-33, lines 935-942).

6. **missing spaces and inconsistent tense exist in the manuscript. improve language.**

We thank the reviewer for this comment. We performed a language and formatting pass to correct spacing issues, tense consistency, and readability throughout the manuscript.

We sincerely thank the Academic Editor and the reviewers for their constructive comments. We believe these revisions have substantially improved the clarity and rigor of our manuscript. All changes have been incorporated into the revised manuscript with track changes, and we hope the revised version is now suitable for publication in PLOS ONE. We would be pleased to address any further questions or suggestions.
