# Supplementary material for: The association between platelet-to-albumin ratio and diabetic peripheral neuropathy: A cross-sectional study in the Chinese population
Source: PLoS One. 2026 May 20;21(5):e0348402. doi: 10.1371/journal.pone.0348402 (PMC13189351; doi:10.1371/journal.pone.0348402)
Supplement: Attachment — Submitted filename: Response_to_Reviewers_auresp_2.docx [file pone.0348402.s006.docx]

**Manuscript ID:** PONE-D-25-53697R1

**Manuscript:** *The association between Platelet-to-albumin ratio and Diabetic peripheral neuropathy: A cross-sectional study in the Chinese population*

Dear Academic Editor and Reviewers,

We sincerely thank you for your careful evaluation of our manuscript and for the constructive comments and suggestions. We have revised the manuscript thoroughly in response to all concerns raised. These revisions have substantially improved the clarity, methodological rigor, and interpretation of the study. All page and line numbers reported below refer to the “Revised Manuscript with Track Changes”, text modifications are marked in red font, unless otherwise specified.

**Response to Reviewer #3**

**Reviewer #3: I have carefully reviewed the manuscript entitled “The association between Platelet-to-albumin ratio and Diabetic peripheral neuropathy: A cross-sectional study in the Chinese population”. While the topic is clinically relevant, there are several major methodological and reporting issues that prevent acceptance of the manuscript in its current form.**

**Q1: Study design vs. claims:**

**The study is described as cross-sectional, yet the introduction and discussion suggest predictive or causal interpretations of PAR for diabetic complications. A cross-sectional design does not allow for temporality or risk prediction. Claims regarding the predictive value of PAR must be removed or appropriately moderated.**

We thank the reviewer for this important comment. We agree that, because this study is cross-sectional, causal inference and prediction of future risk are not appropriate. Accordingly, we carefully revised the manuscript to remove or moderate language implying predictive or causal interpretations. Specifically, we revised the Abstract, Results, Discussion, and Conclusion to consistently describe PAR as being **associated with prevalent DPN**, rather than as a predictor or causal factor. We also revised the ROC-related wording to refer to the **discriminative ability** of PAR for distinguishing patients with and without DPN, rather than its predictive value. In the Discussion and Conclusion, we further clarified that PAR should be interpreted as a **complementary associated marker** rather than a predictive, causal, or standalone clinical tool. In addition, we strengthened the limitations section by explicitly stating that the cross-sectional design precludes causal inference and does not establish temporality.

**Q2: Registry and sample selection:**

1) **The manuscript states that data were derived from the national MMC registry. However, a substantial proportion of patients (967 out of ~3700) did not have diabetes, suggesting that the registry was not specifically designed for T2DM patients.**

**The total number of patients and geographic coverage do not support the claim of a national registry. This raises serious concerns about the representativeness and generalizability of the sample.**

We sincerely thank the reviewer for this insightful comment regarding the description of the MMC database and the representativeness of our study population. First, the Metabolic Management Center (MMC) is a standardized metabolic disease management network initiated in 2016 by the Chinese Diabetes Society of the Chinese Medical Doctor Association and led by Academician Ning Guang. The MMC model aims to provide standardized diagnosis and management of metabolic diseases such as diabetes through an integrated “one center, one-stop service, one standard” approach. By 2019, more than 700 hospitals across 30 provincial administrative regions in China had established MMC centers, collectively managing over 220,000 patients with metabolic diseases. Importantly, the MMC database includes patients with various metabolic conditions, including diabetes, prediabetes, and individuals undergoing metabolic screening. Therefore, the presence of non-diabetic individuals in the initial dataset is expected. In the present study, only patients with confirmed type 2 diabetes mellitus (T2DM) were included in the final analysis after applying predefined inclusion and exclusion criteria, as described in the Methods section.

Thus, although the MMC network is a nationwide standardized management system, our analysis was conducted using data from a single MMC center rather than the entire national MMC registry. To avoid potential misunderstanding, we have revised the manuscript to clarify this point. Specifically: “The study was a cross-sectional analysis based on data obtained from the National Metabolic Management Centre (MMC) program at the Affiliated Hospital of Southwest Medical University, a participating center in the nationwide MMC network in China.” (See Revised Manuscript with Track Changes, **page 5, lines 155–158; page 29, lines 1253–1255**).

We have also added a statement in the Discussion section acknowledging that the single-center design may limit the generalizability of the findings. (See Revised Manuscript with Track Changes: “Additionally, although the MMC network is implemented nationwide, the present analysis was based on data from a single participating center, which may limit the generalizability of the findings.” **page 25, lines 1139–1141**).

**3) The inclusion/exclusion criteria are inconsistently reported, and there is repetition in the text, which reduces clarity.**

We thank the reviewer for pointing out the lack of clarity in the reporting of inclusion and exclusion criteria. In the revised manuscript, we have streamlined the description to avoid repetition. Specifically, redundant statements such as “Only participants with confirmed T2DM were included” were removed because this criterion had already been defined in the inclusion criteria. In addition, exclusion criteria related to non-diabetic individuals were deleted to avoid logical overlap with the inclusion criteria. The description of participant selection was simplified, and the recruitment period was unified across the manuscript to ensure consistency. These revisions improve the clarity of the study design. (See Revised Manuscript with Track Changes: “Eligible participants were adults (age≥18 years) with confirmed T2DM according to the American Diabetes Association (ADA) "Standards of Care in Diabetes"; Exclusion criteria:1) type 1 diabetes, gestational diabetes and other special types of diabetes; 2)Use of oral antiplatelet and anticoagulant drugs;3)History of infection within 3 months, severe diabetic foot complications, or severe liver or kidney disease; 4)Patients with malignant tumors, hematological disorders; 5)Missing data.” page 6, lines 176–182).

**Q3: Ethical considerations:**

**The manuscript indicates that “all participants provided written informed consent for this study.” Given that the analysis is based on registry data, it is unclear whether the consent was obtained specifically for this study or as part of enrollment in the registry.**

We thank the reviewer for this comment. We clarified that written informed consent was obtained at the time of enrollment in the MMC program, and that this consent included permission for the use of anonymized clinical data for research purposes. We revised the Ethics Approval section. (See Revised Manuscript with Track Changes: “All participants provided written informed consent at the time of enrollment in the Metabolic Management Center (MMC) program, which included permission for the use of their anonymized clinical data for research purposes.”, **page 6, lines 186–188**)

**Q4: Outcome measurement and variable definition:**

**1)The description of laboratory measurements is unclear. For example, 2-hour plasma glucose (2h PG) is reported to be measured from fasting blood samples, which is inconsistent with standard OGTT procedures.**

We thank the reviewer for pointing out this ambiguity. In the MMC database, fasting plasma glucose (FPG) and 2-hour plasma glucose (2h PG) are measured using venous blood samples collected during a standard oral glucose tolerance test (OGTT). We have revised the manuscript to clarify this description. (See Revised Manuscript with Track Changes: “FPG and 2h PG were measured using venous blood samples collected during a standard OGTT, with samples obtained after an overnight fast and again 2 hours after glucose ingestion. Additional morning fasting blood samples were collected from each patient after an 8-hour fast.”, **page 8, lines 249–252**)

**2)It is not clear whether patients were assessed only once or followed over time. The timing of laboratory measurements (baseline, most recent, or averaged) must be specified.**

We thank the reviewer for this important point. We clarified that although patients enrolled in the MMC program may receive regular follow-up as part of routine clinical management, the present study was designed as a cross-sectional analysis. Accordingly, only measurements obtained at a single time point were used in the present analysis. We added this clarification to the Methods section to avoid misunderstanding regarding temporality or repeated measures use.(See Revised Manuscript with Track Changes: “Patients in the MMC program receive continuous follow-up as part of routine clinical management. For this cross-sectional analysis, only clinical and laboratory data obtained at the first MMC visit were used.”, **page 5, lines 160–163**)

**Q5: Statistical analysis:**

**The multivariable model includes a very large number of covariates, many of which are highly collinear (e.g., FPG and HbA1c; SBP and DBP; Hb, RBC, HCT; lipid profile variables). There is no report of collinearity diagnostics. This may lead to unstable and uninterpretable regression coefficients. Spearman correlation is incorrectly used to assess associations between PAR and a binary outcome (DPN). Appropriate methods would include logistic regression or nonparametric comparison tests.**

We sincerely thank the reviewer for these valuable comments. Following the reviewer’s suggestions, we carefully re-evaluated the statistical analysis and made several revisions to improve the robustness and appropriateness of the methods.

1. Multicollinearity diagnostics

We agree that including multiple correlated clinical variables may lead to multicollinearity and unstable regression coefficients. Therefore, **multicollinearity diagnostics were performed prior to constructing the multivariable logistic regression models**. Variance inflation factors (VIFs) were calculated for all candidate variables using SPSS.Variables with **VIF > 5** were considered to have potential multicollinearity and were excluded from the final model. For example, **serum creatinine (Cr)** showed multicollinearity with **eGFR** and was therefore removed. After this screening process, **all variables retained in the final model had VIF values below 5**, indicating acceptable levels of multicollinearity.

| Model | | Unstandardised coefficient | | Standardised coefficient | t | P-value | Collinearity statistics | |
| --- | --- | --- | --- | --- | --- | --- | --- | --- |
|  |  | B | standard error | Beta |  |  | tolerance | VIF |
|  | (Constant) | -0.289 | 0.261 |  | -1.107 | 0.268 |  |  |
|  | Age | -0.004 | 0.001 | -0.097 | -2.998 | 0.003 | 0.688 | 1.453 |
|  | History of antihypertensive drugs | -0.162 | 0.034 | -0.138 | -4.743 | 0.000 | 0.856 | 1.169 |
|  | duration | 0.001 | 0.000 | 0.087 | 2.910 | 0.004 | 0.812 | 1.232 |
|  | SBP | 0.002 | 0.001 | 0.089 | 2.687 | 0.007 | 0.659 | 1.517 |
|  | Heart rate | 0.004 | 0.001 | 0.118 | 4.218 | 0.000 | 0.931 | 1.074 |
|  | BMI | -0.013 | 0.004 | -0.098 | -3.412 | 0.001 | 0.875 | 1.143 |
|  | HAb1C | 0.039 | 0.006 | 0.197 | 6.847 | 0.000 | 0.869 | 1.150 |
|  | WBC | -0.002 | 0.007 | -0.006 | -0.215 | 0.830 | 0.820 | 1.220 |
|  | MCV | -0.001 | 0.002 | -0.018 | -0.659 | 0.510 | 0.929 | 1.077 |
|  | ALT | -0.001 | 0.001 | -0.063 | -1.378 | 0.168 | 0.344 | 2.911 |
|  | AST | 0.001 | 0.001 | 0.030 | 0.663 | 0.508 | 0.349 | 2.867 |
|  | BUN | 0.018 | 0.007 | 0.071 | 2.448 | 0.015 | 0.858 | 1.165 |
|  | LDLc | -0.006 | 0.013 | -0.014 | -0.485 | 0.628 | 0.924 | 1.082 |
|  | logACR | 0.044 | 0.009 | 0.156 | 4.940 | 0.000 | 0.723 | 1.383 |
|  | PAR | 0.029 | 0.009 | 0.094 | 3.084 | 0.002 | 0.775 | 1.291 |
| a. Dependent variable: DPN; Average VIF=1.449 | | | | | | | | |

The revised multivariable logistic regression results are presented in the **updated Table 2 and Table 3**(**page 16, lines 401; page 18, lines 684**;).

**2. Removal of Spearman correlation analysis**

We appreciate the reviewer’s comment regarding the inappropriate use of Spearman correlation for a binary outcome. Following this suggestion, the Spearman correlation analysis has been removed from the revised manuscript. Associations between PAR and DPN are now evaluated exclusively using logistic regression models (Table 2) and quartile-based analyses (Table 3), which are more appropriate for a binary outcome.

**3. Revision of the statistical analysis section**

The **Statistical Analysis** section of the manuscript has been revised to clearly describe:

- the procedure for **multicollinearity diagnostics**
- the **variable selection strategy**
- the use of **logistic regression models** for binary outcomes

These revisions improve the methodological rigor and transparency of the statistical analysis. (See Revised Manuscript with Track Changes: “. Multicollinearity was assessed using variance inflation factors (VIF) derived from a linear regression model including all candidate predictors; redundant covariates with VIF > 5 were excluded. Variables that represent clinical manifestations or consequences of diabetic peripheral neuropathy, such as a history of diabetic foot or nerve conduction study results, were not included as covariates, as they are downstream outcomes of neuropathy rather than independent confounding factors. ”, **page 5, lines 160–163**).

**Response to Reviewer#5**

**Reviewer #5: This manuscript represents methodologically sound research. The authors made significant changes in the revised version, correctly repositioning PAR as an associated marker rather than a screening tool.**

1. **Authors should rightly conclude clinical implications.**

We thank the reviewer for this important comment. We revised the Discussion and Conclusion to ensure that the clinical implications are stated more appropriately and conservatively. In the revised manuscript, we now emphasize that PAR is an accessible and inexpensive laboratory marker associated with prevalent DPN, but that its clinical utility is limited by the modest effect size and limited discriminative performance observed in our ROC analysis. We explicitly state that PAR should not be interpreted as a standalone screening, diagnostic, or predictive tool. Instead, it may provide complementary information regarding the inflammatory and nutritional status of patients with DPN. (See Revised Manuscript with Track Changes: **4. Discussion; page 5, lines 914–1194**)

2. **Authors should address why only T4 shows significance despite "linear" spline.**

We appreciate this insightful comment. We revised the Discussion to address this point more directly. Specifically, we now explain that the restricted cubic spline (RCS) model evaluates the overall continuous association between PAR and DPN across the full distribution of PAR, whereas the quartile-based analysis compares categorized groups against a reference group. Categorizing a continuous variable into quartiles may reduce statistical power and make smaller differences less likely to reach statistical significance. In our analysis, the odds ratios for T2 and T3 were directionally consistent with a positive association, but only the contrast between T4 and T1 was large enough to reach statistical significance. We therefore clarify that the spline and quartile findings are complementary rather than contradictory. (See Revised Manuscript with Track Changes: “Restricted cubic spline analyses supported an overall positive linear association between PAR and the prevalence of DPN, while quartile-based analyses suggested that the association became statistically more apparent at higher PAR levels. The quartile-based analysis and spline modeling were complementary rather than contradictory. This may reflect the loss of statistical power after categorizing a continuous variable, such that smaller between-group differences in T2 and T3 did not reach statistical significance. While the spline analysis suggested a generally positive continuous trend between PAR and prevalent DPN, the quartile analysis indicated that the association was more pronounced among individuals with higher PAR levels.”; **page 21, lines 920–929**)

**3. Authors should examine the robustness to different DPN definitions.**

We thank the reviewer for this thoughtful suggestion. We carefully evaluated the feasibility of conducting sensitivity analyses using alternative definitions of DPN. However, in the MMC database used for the present study, the available related variables were limited to the final conclusion of sensory nerve conduction testing, brachial-ankle pulse wave velocity (baPWV), and ankle-brachial index (ABI). The database did not contain sufficient raw neurological examination components or detailed electrophysiological parameters to reconstruct independent alternative diagnostic definitions of DPN. In addition, baPWV and ABI primarily reflect vascular status rather than neuropathy and therefore were not considered appropriate substitutes for DPN classification. To avoid introducing misclassification or methodological bias, we did not perform sensitivity analyses based on alternative DPN definitions. We have acknowledged this as a limitation in the revised Discussion section.
